# Incomplete homonymous hemianopsia progressing to junctional scotoma due to a large internal carotid artery aneurysm: A case report

**DOI:** 10.1002/ccr3.8872

**Published:** 2024-04-30

**Authors:** Yuki Takagi, Kenta Hozumi, Sho Yokoyama, Yoshimi Yokoyama, Tatusi Kaga

**Affiliations:** ^1^ Department of Ophthalmology Japan Community Healthcare Organization Chukyo Hospital Nagoya Japan

**Keywords:** compression optic neuropathy, glaucoma, incomplete homonymous hemianopsia, internal carotid artery aneurysm, junctional scotoma

## Abstract

Visual field disorders caused by cerebral aneurysms are diverse, nonspecific, and vary in their degree of compression. They should be distinguished from those caused by other common diseases, such as glaucoma.

## 1 INTRODUCTION

Cerebral aneurysms may cause a variety of visual field defects, such as junctional scotoma, bilateral hemianopsia, and monocular central visual defects, depending on the site of origin, direction of extension, and size of the aneurysm.^1^, ^2^, ^3^, ^4^, ^5^, ^6^, ^7^ Jefferson classified visual fields defects caused by cerebral aneurysms into three groups: (1) scotomatous homolateral eye, with an overlying homonymous defect in the nasal field of that eye and in the temporal field of the other eye; (2) bitemporal defects, clearly aneurysmal because of inferior quadrant loss as the earliest sign, suddenness of development, or ocular muscle palsies, as well as pain; and (3) nasal hemianopsia in the ipsilateral eye.^4^ He also reported that junctional scotoma is a characteristic of internal carotid artery aneurysms.^4^ Farris et al. reported nasal visual field abnormalities as a sign of aneurysm.^1^

Although numerous studies have reported visual field defects caused by cerebral aneurysms, to the best of our knowledge, changes in the visual field abnormality have rarely been reported, with no reports of junctional scotoma developing from homonymous hemianopsia. Herein, we report a case in which a large, unruptured internal carotid artery aneurysm caused incomplete left homonymous hemianopsia, which rapidly progressed to junctional scotoma.

## 2 CASE HISTORY AND EXAMINATION

**FIGURE 1:**
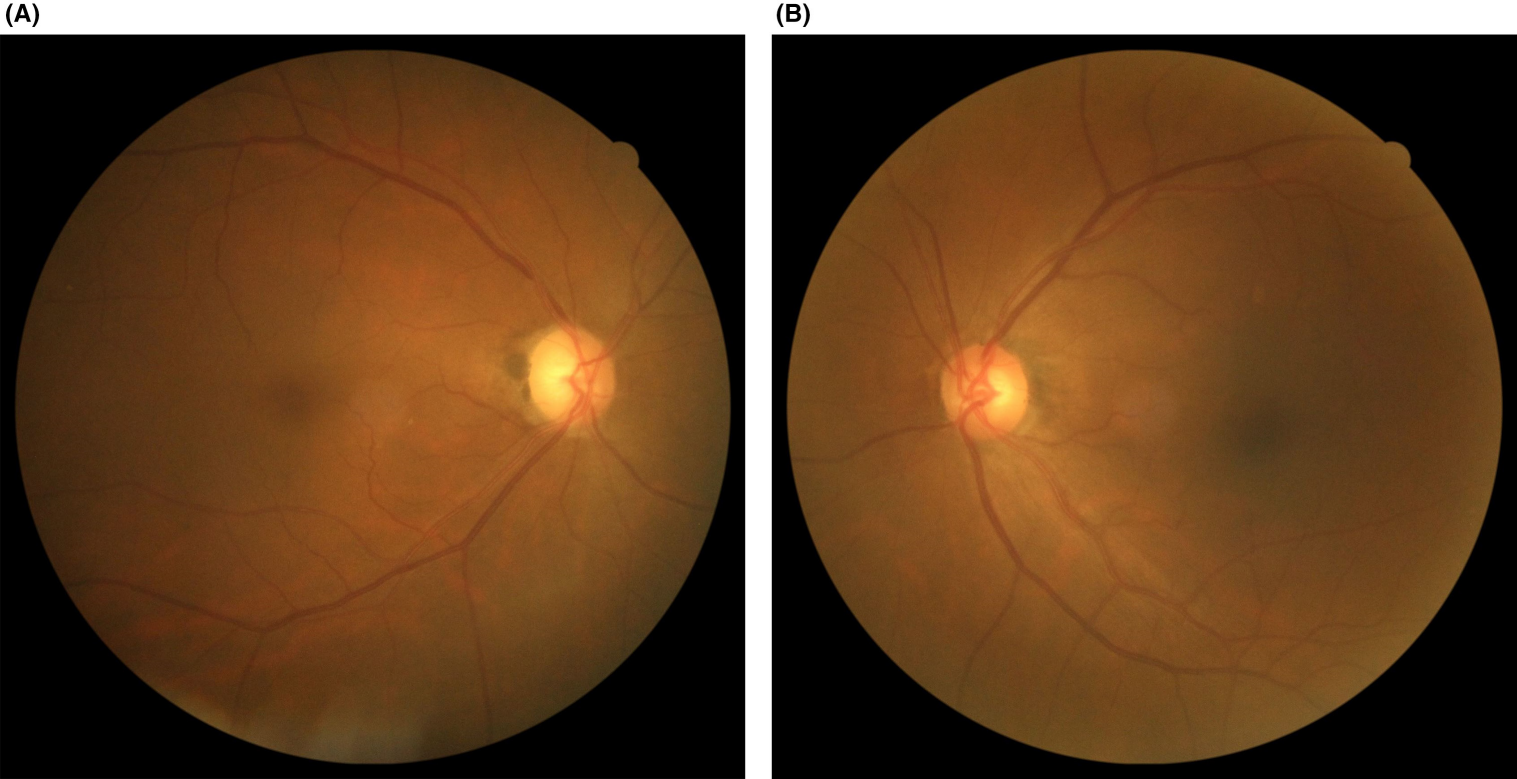
Fundus examination findings during the initial visit at our hospital. (A) There is pallor on the temporal side of the optic nerve papilla in the right eye. (B) No abnormalities is seen in the left eye.

A 65‐year‐old woman visited an ophthalmology clinic in July 2022 due to difficulty seeing with her right eye. Glaucoma was suspected and antiglaucoma eye drops were prescribed. Four months later, in November 2022, she visited our hospital. The best corrected visual acuity was 20/100 in the right eye and 20/20 in the left, and the critical fusion frequency was 20 and 39 Hz, respectively. The pupil diameter did not differ between the right and left eyes; however, the right eye was positive for relative afferent pupillary defect. There was no obvious ocular motility disorder. Fundus examination revealed pallor on the temporal side of the optic nerve papilla in the right eye (Figure 1A,B).

## 3 DIFFERENTIAL DIAGNOSIS, INVESTIGATIONS, AND TREATMENT

**FIGURE 2:**
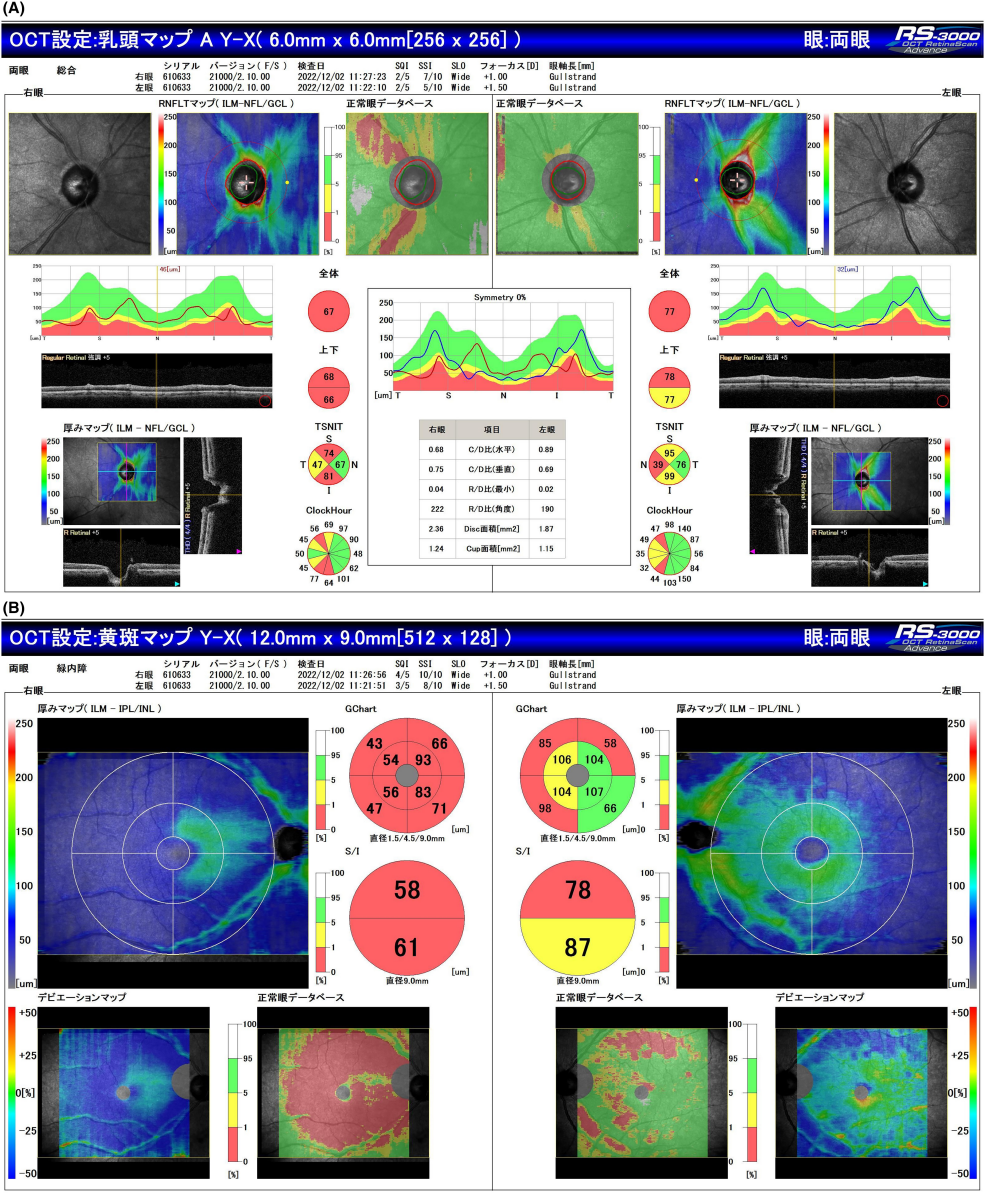
Optical coherence tomography (OCT) findings. (A) A “tie” atrophy of the nerve fiber layer of the right eye is observed, whereas no abnormality is seen in the left eye. (B) Diffuse thinning of the ganglion cell complex is visualized in the right eye and predominant nasal atrophy in the left eye.

**FIGURE 3:**
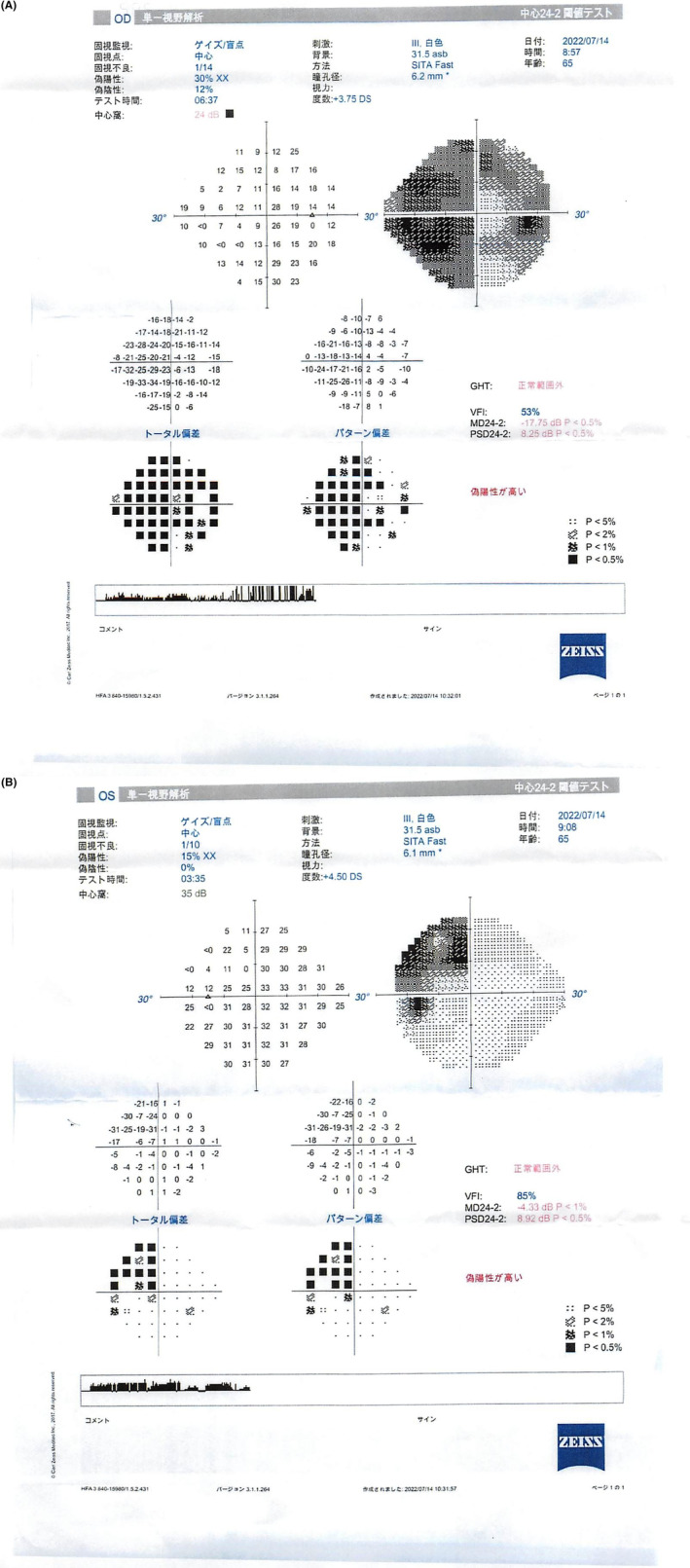
Humphrey field analyzer (HFA) results from July 2022. (A) There is a visible nasal‐predominant visual field defect in the right eye and (B) upper temporal visual field defect in the left eye, with incomplete left homonymous hemianopia.

**FIGURE 4:**
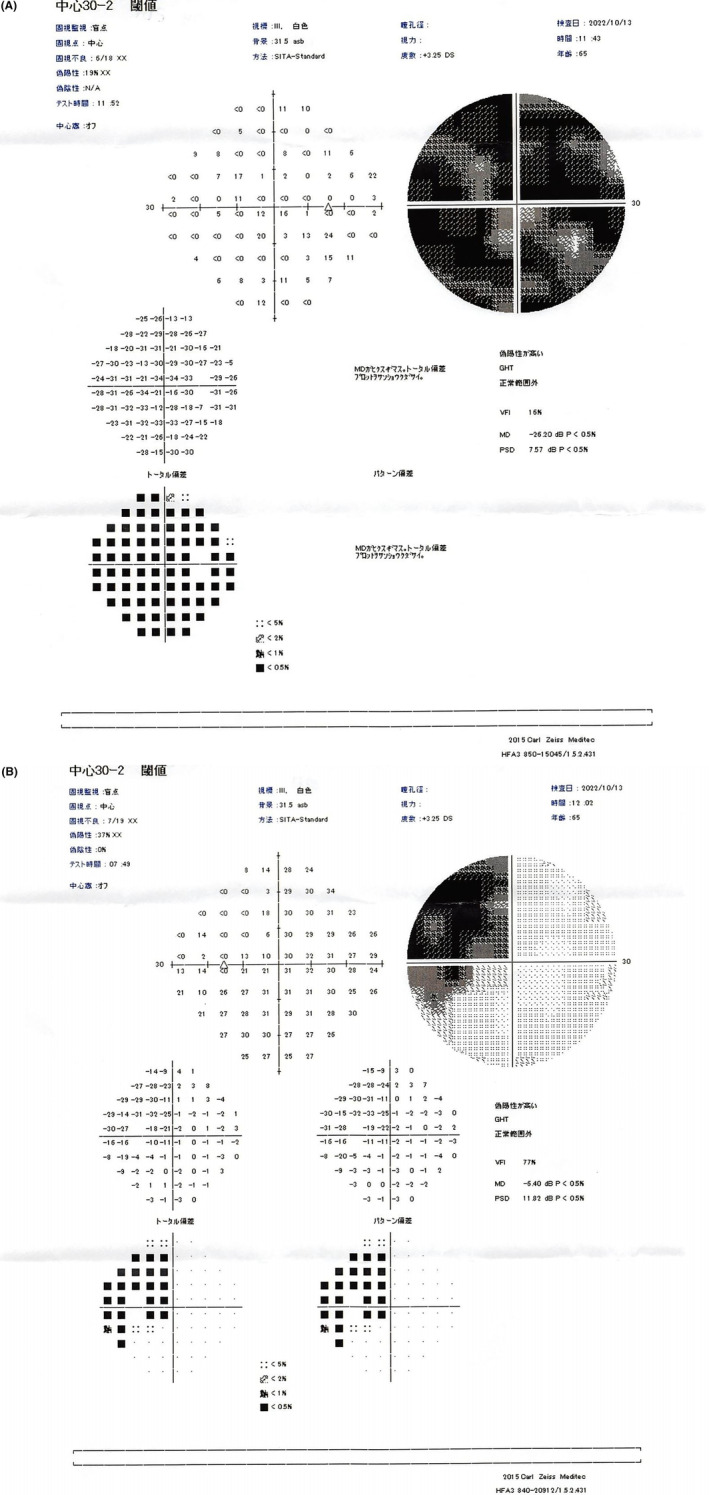
Humphrey field analyzer (HFA) results from October 2022. (A) There is rapid progression of the visual field defects in the right eye and (B) newly developed central visual field defects, as well as worsening of the upper temporal hemianopsia in the left eye.

Optical coherence tomography (OCT) revealed a “tie” atrophy of the nerve fiber layer of the right eye and predominant nasal atrophy in the left eye (Figure 2A,B). Visual field evaluation using a Humphrey field analyzer (HFA) at the previous ophthalmic clinic revealed a nasal‐predominant visual field defect in the right eye and upper temporal visual field defect in the left eye, with incomplete left homonymous hemianopia (Figure 3A,B). The HFA evaluation performed at our hospital showed rapid progression of the visual field defects in the right eye, with development of central visual field defects, as well as worsening of the upper temporal hemianopsia in the left eye (Figure 4A,B). This visual field abnormality was considered consistent with the OCT findings.

**FIGURE 5:**
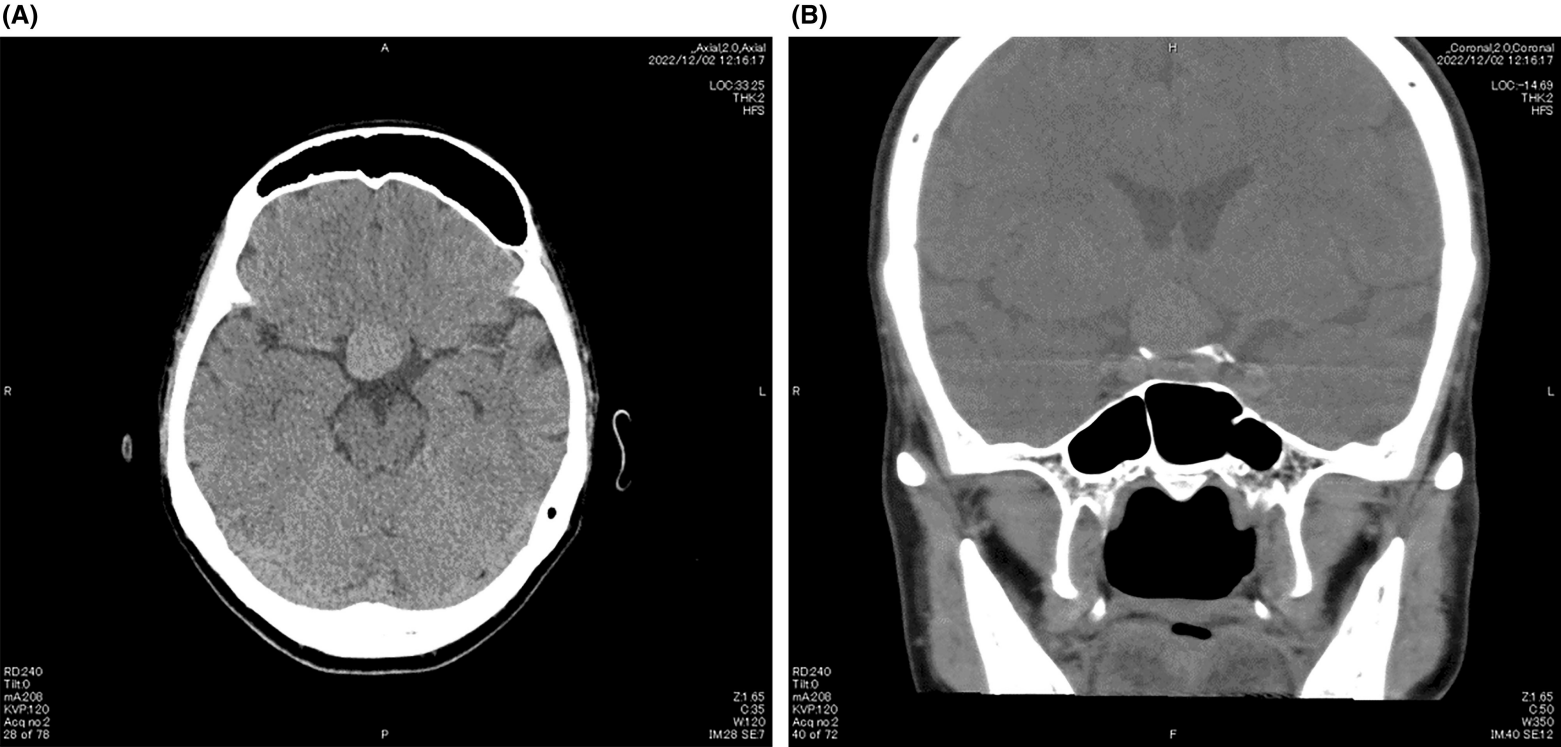
Brain computed tomography findings. (A) Axial and (B) sagittal images showing a large mass slightly to the right above the sella.

**FIGURE 6:**
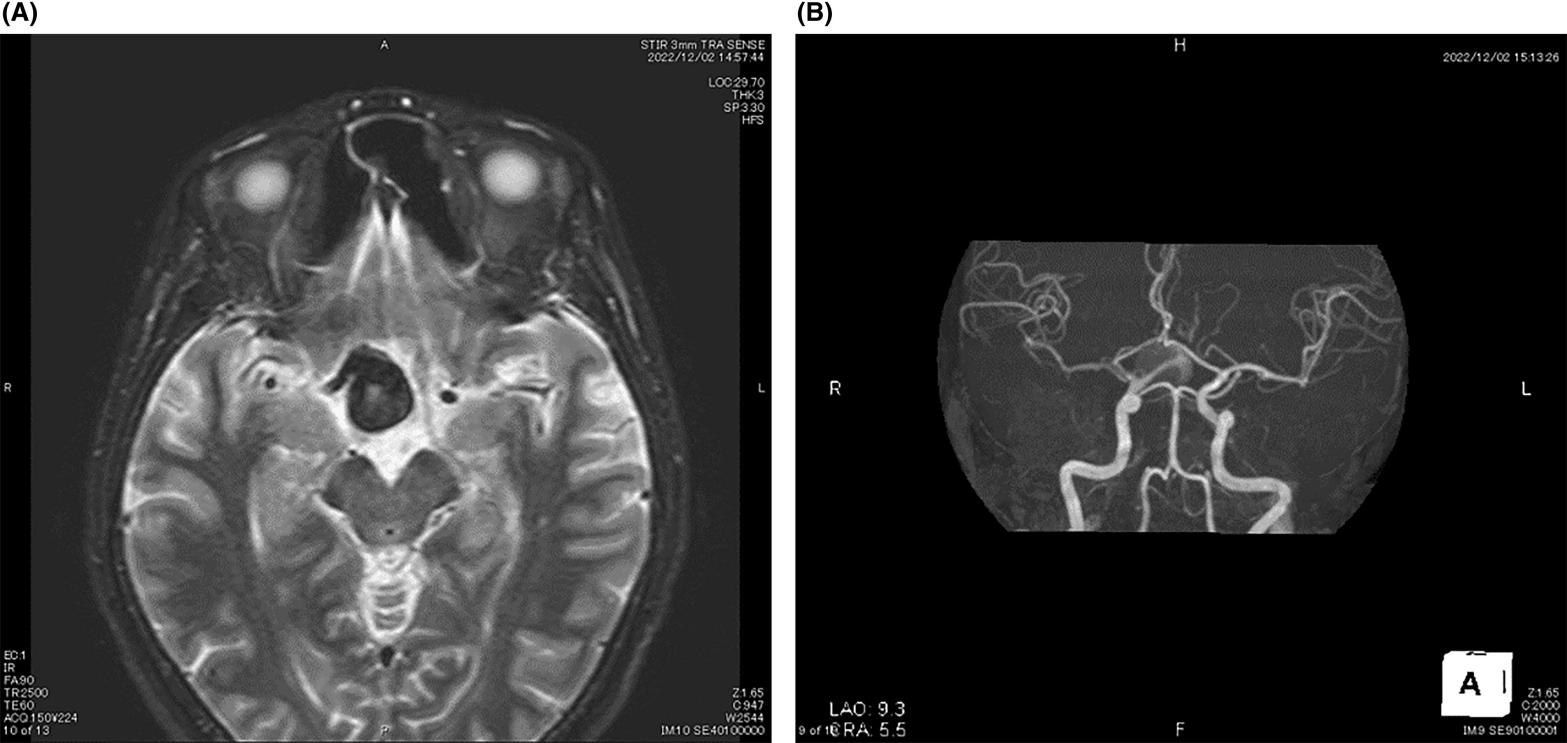
Brain magnetic resonance imaging findings. (A) Axial image showing a large unruptured aneurysm sized 18 × 17 mm extending from the right internal carotid artery. (B) Magnetic resonance angiography image showing a large unruptured aneurysm originating from the right internal carotid artery.

Based on the visual field defects, we suspected junctional scotoma and performed brain computed tomography, which showed a large mass slightly to the right above the sella (Figure 5A,B), as well as brain magnetic resonance imaging, which showed a large unruptured aneurysm sized 18 × 17 mm extending from the right internal carotid artery (Figure 6A,B). The aneurysm caused compression of the right optic nerve to the anterior part of the optic chiasm from the inferior temporal side, with the optic chiasm deviated to the left from its normal position. The patient was treated by aneurysmal clipping at the neurosurgery department.

## 4 OUTCOME AND FOLLOW‐UP

**FIGURE 7:**
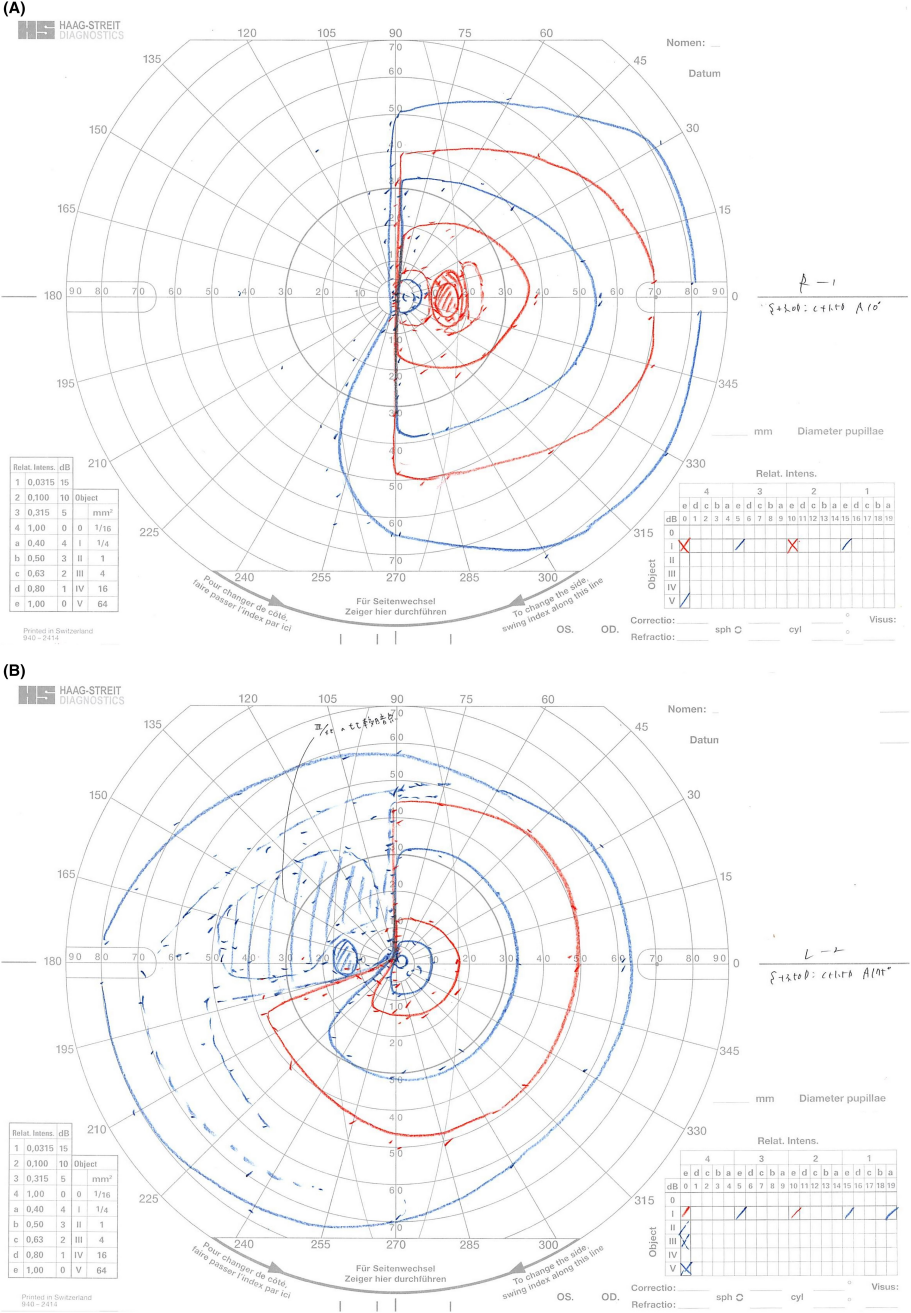
Goldmann perimetry findings 2 weeks after clipping surgery. (A) There is nasal hemianopsia in the right eye and (B) upper temporal visual field defect in the left eye.

**FIGURE 8:**
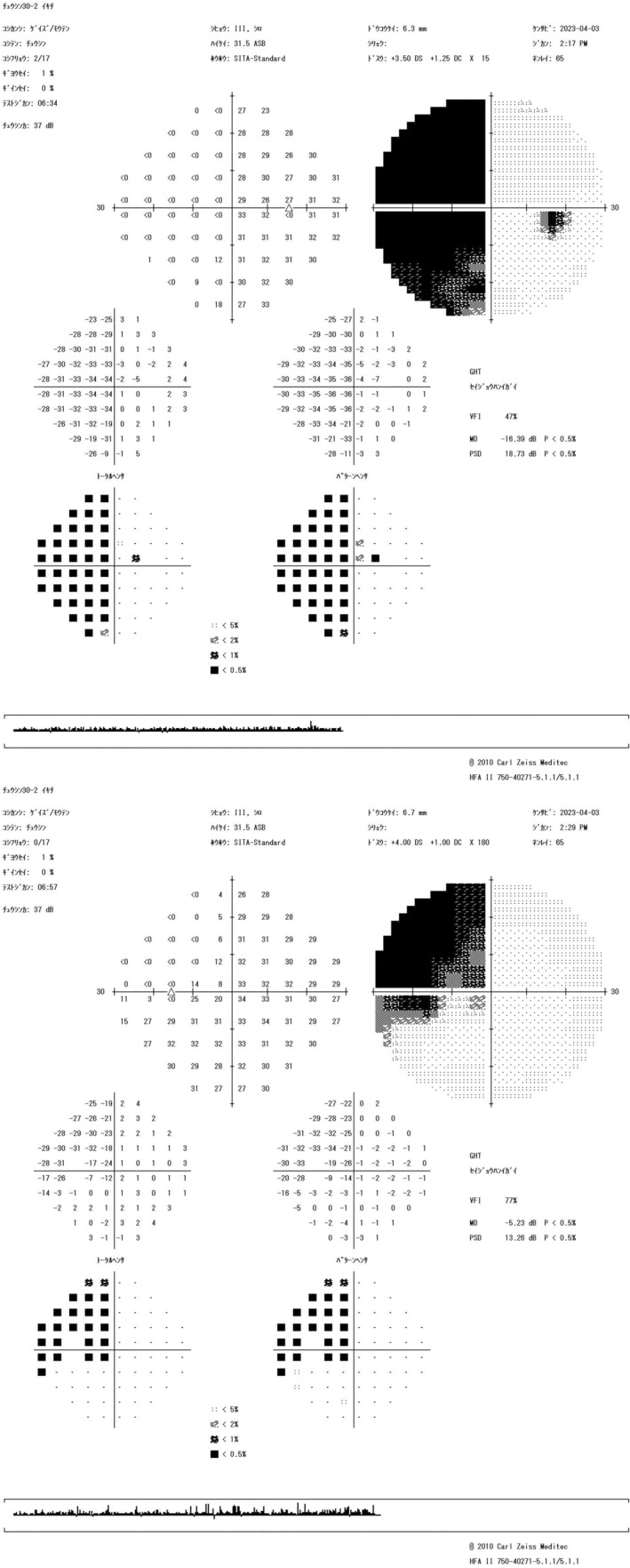
Humphrey field analyzer (HFA) findings 4 months after the clipping surgery. (A) There is nasal hemianopsia in the right eye and (B) upper temporal visual field defect in the left eye.

At the follow‐up ophthalmologic examination performed 2 weeks after the surgery, the visual acuity and critical fusion frequency of the right eye improved to 20/16 and 27.6 Hz, respectively. Goldmann perimetry performed at that time showed nasal hemianopsia in the right eye and upper temporal visual field defect in the left eye (Figure 7A,B). HFA evaluation performed 4 months postoperatively showed similar results (Figure 8A,B).

## 5 DISCUSSION

Junctional scotoma is characterized by central visual field defect in the affected eye and upper temporal hemianopsia in the fellow eye. Although it is most commonly caused by pituitary tumors,^8^, ^9^ it has also been reported in cases of cerebral aneurysms.^1^, ^2^, ^3^, ^4^, ^5^, ^6^, ^7^ A prior study suggested that cerebral aneurysm should be suspected in cases of ipsilateral nasal visual field defects.^1^ Notably, when the optic chiasm is compressed from the lateral side by a cerebral aneurysm, nerve fibers in the temporal retinal area (nasal visual field) running in this region are likely to be affected. Indeed, in the present case, the nasal visual field was initially affected. In another study, junctional scotoma was suggested as a characteristic visual field defect caused by internal carotid artery aneurysms.^4^ This is also consistent with the findings in our case, as the visual field defect at the time of diagnosis was junctional scotoma and the cause was an internal carotid artery aneurysm.

However, as per the examination findings from the ophthalmic clinic where our patient was initially evaluated, she had nasal‐predominant visual field defect in the right eye and upper temporal visual field defect in the left eye, with incomplete left homonymous hemianopsia. Based on this history, the following was the suggested mechanism of the visual field defect in this case. Initially, the pressure from the internal carotid artery aneurysm on the anterior lateral side of the optic chiasm resulted in damage to the nerve fibers in the temporal retinal area (nasal visual field) of the affected eye and the inferior nasal retinal area (upper temporal visual field) of the contralateral eye. The progressive compression further caused right optic nerve damage, with changes to the nerve fibers in the nasal retinal area (temporal visual field) on the affected eye, resulting in progression of the visual field defect with development of central visual field defect. We believe that the damage caused by the internal carotid artery aneurysm to the anterior part of the optic chiasm preceded the damage to the optic nerve on the affected side because if compression of the optic nerve on the affected side was the initial change, the central visual field defect on the affected eye would have been more progressed than the upper temporal field defect of the contralateral eye. In addition, postoperatively, the temporal visual field defect and visual acuity in the affected eye improved, whereas the upper temporal visual field defect in the contralateral eye persisted. The postoperative visual field test results may suggest that the damage to the nerve fibers in the inferior retinal area of the contralateral eye preceded that to the nasal retinal area of the affected eye. In a prior study, a large internal carotid artery aneurysm caused visual field defects in the contralateral upper temporal side and central visual field defects in the affected eye, while the upper temporal visual field in the affected eye remained intact.^10^ This suggests that the nerve fibers in the inferior nasal retinal area of the affected eye are unlikely to be affected only by damage to the anterior part of the optic chiasm, which is also supported by the course of findings in the present case.

Junctional scotoma was once thought to be caused by Willbrand's knee disorder^11^; however, the existence of Willbrand's knee was ruled out.^12^ In a study examining visual field defects caused by optic chiasm disorders due to brain tumors, the tumor size tended to be larger in cases with junctional scotoma than in cases with other visual field defects.^9^ This was assumed to be due to the extensive compression and damage caused by large tumors not only to the optic chiasm but also to the optic nerve. In the present case, the large internal carotid artery aneurysm compressed and damaged not only the optic chiasm but also the optic nerve, which is consistent with the previously reported findings. In addition, if Willbrand's knee compression had caused the visual field abnormality in the present case, as the nerve fibers in the contralateral inferior nasal retinal area are located medial to the affected optic nerve, neuropathy would have occurred at the same time as in the nerve fibers in the affected nasal retinal area, which is inconsistent with the course in this case. Therefore, our case does not seem to support the existence of Willbrand's knee.

A specific feature of the current case is the progression of visual field defects. Conway reported a case of visual field defect changes during follow‐up,^13^ with temporary improvement in the visual field defect, which was assumed to be due to the relieved pressure by aneurysm thrombosis. In our case, the visual field defect deteriorated with time, which is consistent with aneurysmal enlargement and progressive neuropathy. Nevertheless, the fact that the visual field defect changed as the compression progressed suggests that the visual field defect in the hypothetically mildly compressed state would also have been different. If the compression was mild, it is possible that the visual field defect would have been milder than that determined at the initial evaluation and more similar to that seen in glaucoma.

The present study had several limitations. First, only 24–2 evaluation was performed prior to clipping surgery, while Goldmann perimetry was not performed. As a result, the details of the peripheral vision defect and postoperative recovery were unknown. Second, we believe that the existing optic nerve damage was already advanced at the time of the initial evaluation at the other ophthalmology clinic. It is unclear what type of visual field defect would have been present if a visual field test had been performed at an earlier stage.

Visual field disorders caused by cerebral aneurysms are diverse, nonspecific, and vary in their degree of compression. They should be distinguished from those caused by other common diseases, such as glaucoma. This case suggests that some cases of cerebral aneurysm may be misidentified as glaucoma. If sudden visual field changes occur, it is important to perform intracranial examinations.

## AUTHOR CONTRIBUTIONS

**Yuki Takagi:** Conceptualization; investigation; visualization; writing – original draft. **Kenta Hozumi:** Writing – review and editing. **Sho Yokoyama:** Supervision; visualization; writing – review and editing. **Yoshimi Yokoyama:** Writing – review and editing. **Tatusi Kaga:** Supervision.

## FUNDING INFORMATION

This study did not receive any specific grants from funding agencies in the public, commercial, or non‐profit sectors.

## CONFLICT OF INTEREST STATEMENT

The authors have no conflict of interest to declare.

## CONSENT

Written informed consent was obtained from the patient to publish this report in accordance with the journalɧs patient consent policy.

## Data Availability

Data sharing is not applicable to this article as no datasets were generated or analyzed during the current study.
